# Revised cutoff values of ALT and HBV DNA level can better differentiate HBeAg (-) chronic inactive HBV patients from active carriers

**DOI:** 10.1186/1743-422X-8-86

**Published:** 2011-02-27

**Authors:** Bushra Ijaz, Waqar Ahmad, Fouzia T Javed, Sana Gull, Sajida Hassan

**Affiliations:** 1 Applied and Functional Genomics lab, Centre of Excellence in Molecular Biology, University of the Punjab, Lahore, Pakistan; 2 Department of Pathology, Jinnah Hospital, Lahore, Pakistan

## Abstract

**Background and Aims:**

ELISA is still used as primary test for diagnosis HBV disease. However, ELISA-positive patients were marked as HBV inactive after confirmation with PCR and vice versa. Our aim was to assess the performance of new cut-off value of ALT, HBV DNA load and significance of AST as screening tool for HBeAg (-) chronic active or inactive patients in Pakistani population.

**Materials and methods:**

In a cross-sectional, cohort study, 567 HBeAg (-) patients followed for one year were selected. Patients with persistent elevated ALT than normal and HBV DNA ≥ 100,000 copies/mL were taken as active chronic. Diagnostic values for ALT, AST and HBV DNA load in HBV HBeAg (-) chronic active and inactive patients compared using receiver operation characteristic (ROC) curves.

**Results:**

Of 567 HBeAg (-) patients, 228 were classified as chronic inactive and 339 as active. HBV infection was dominant in male. Serum ALT, AST and HBV DNA levels showed significant and high AUROC to differentiate chronic HBeAg (-) inactive patients from active. AUROC for Serum ALT, AST and HBV DNA were observed 0.997, 0.969 and 1.000, respectively. For revised cut off value for ALT (30 IU/L for male and 19 IU/L for female) and HBV DNA load ≥100,000 copies/mL, a PPV of 97%, NPV of 94%, a sensitivity of 98%, and a specificity of 92% was observed to discriminate active carriers from inactive carriers. We also observed 93.5% specificity, 83.1% sensitivity, 82% PPV and 89.5% NPV for AST ≤20 IU/L to differentiate inactive carriers from active ones in our study group.

**Conclusions:**

Revised cut off value of ALT and NIH derived HBV DNA value can better discriminate between HBeAg (-) chronic active and inactive patients.

## Introduction

Almost 170-200 million of the world population is infected with HBV, leading to world's most common cancer "Hepatocellular carcinoma (HCC)", causing nearly one million deaths per year. Approximately, 20% of chronic HBV patients have eventually progressed to liver cirrhosis, and some infections have evolved into HCC in a substantial number of patients [1,2].

The most common contradiction in diagnosis of HBV patients is the differentiation of chronic active cases from the inactive carriers, as they share same serological profile. Diagnosis of disease outcome in these patients with PCR and HBV DNA levels assay, and defining the state of infection with these tools is emerging during last decade [3-5]. However, in many countries and regions like United States, Western Europe and other high or middle income countries, ELISA is still used and majority of the positive tests are not confirmed by PCR. It is interesting to note that many HBeAg (-) patients showed presence of chronic active HBV in further screening by PCR and vice versa [6]. To differentiate active chronic HBV from inactive carrier state, an arbitrary serum HBV DNA level of 100000 copies/mL has been proposed by the United States national Institute of health (NIH) [7]. During HBV disease progression, after seroconversion (HBeAg (+) to HBeAg (-), HBeAg consists of two clinical forms; one known as chronic inactive with low persistant aminotransferase levels and HBV DNA levels (≤ 100,000 copies/ml) and second with no HBeAg, high ALT and HBV DNA levels (≥ 100,000 copies/ml). In low-income countries like Pakistan, many patients refused to do PCR and liver biopsy procedure due to poverty and cost of these tests. Beside these challenges, the growing concern is the early detection of viral hepatic disease and liver damage. For this purpose, in routine laboratory tests, elevated alanine aminotransferase (ALT) levels are used as indicators of liver cell injury and as non-invasive diagnostic tests [8]. Elevated AST levels are usually predominant in liver cirrhosis with increased ALT levels [9,10]. During assessment of liver disease due to hepatitis, serum AST and ALT levels are most commonly used serum markers to detect acute and chronic hepatocytes cytotoxicity [11-13]. Now a days, the main emphasis of workers is early detection of liver damage due to chronic HBV, however, there is always questions about the effectiveness of these test because of their low sensitivity [14,15]. Several studies in Italy, China, Korea and Hong Kong showed that ALT levels higher than the normal limits are strongly associated with an increased risk of liver cirrhosis in HBV infected patients [16-19]. Recent studies revealed that in patients with HBeAg (-), high ALT levels greater than 0.5x to the upper limit of normal (ULN) relate to advance fibrosis and ALT > 30 IU/L and 19 IU/L in male and female respectively, with base line HBV DNA levels > 100000 copies/ml; can better differentiate between active chronic HBV patients from inactive chronic carriers [11,12,20-22]. While, Degertekin *et al. *proposed HBV DNA cutoff values of 5000 copies/mL to differentiate between active chronic HBV patients from inactive chronic carriers [23]. These studies indicates ALT level as a reliable serum marker leading to fact that HBV natural history can vary from one population to another [24]. Therefore, we should determine more reliable cutoff value for serum ALT and AST levels to predict active HBV patients according to our population.

The aim of our study was to assess the relationship between HBV DNA load, AST and ALT levels in HBeAg (-) patients, review the performance of serum ALT and HBV DNA levels as the screening tool for liver disease and to find whether new cutoff values of ALT and HBV DNA are able to predict HBV infection in Pakistani population. The need of PCR and invasive procedure liver biopsy may be eradicated if the serum biochemical marker ALT with high positive or negative predictive values of HBeAg (-) patients can be obtained and thus minimize the cost of PCR and therapy time.

## Material and methods

### Patients

Patients of this study were the native Pakistani people referred to Pathology department, Jinnah Hospital, Lahore, Pakistan, for biochemical and serological tests. This retrospective cross-sectional study was carried out from March 2008 to September 2009 with collaboration of National Centre of Excellence in Molecular Biology, University of the Punjab, Lahore, Pakistan. Blood samples (10 mL) collected from each patient tested for HBsAg and HBeAg by ELISA. All patients did not have any history about HBV vaccination or disease infection, and/or other type of hepatitis. The routine liver function tests (LFTs) were estimated for each patient in hospital laboratory. Patients with positive serology and/or positive test for HBV alone and no evidence of liver failure were included in this study. Informed consents were obtained from patients containing their bio data and lab results. This study was approved by the Institutional ethics committee.

### Laboratory assays

Test for HBsAg and HBeAg were done by using ELISA kits (Abbot Diagnostics). Serum ALT and AST levels were measured by using commercially available Hitachi-7600 series automatic analyzer. The normal limits considered for ALT was 40 IU/L and for AST 35 IU/L. Serum HBV DNA was evaluated by using commercially available polymerase chain reaction assay (Amplicor HBV Moniter test; Roche Diagnostic System, Inc., Branchburg, New Jersey) with lower limit of detection 80 copies/mL and accurate range 500-200,000 copies/mL according to manufacturer protocol.

Selected patients were HBeAg (-) with base line ALT determined at first visit. Patients ALT levels were determined four times every three months. Patients were divided into two groups as inactive (A) and active (B) chronic carriers based on HBeAg absence, liver diagnosis by ultrsonography, persistent ALT levels and HBV DNA load. Patients with ≥ 100000 HBV DNA copies/mL and continual elevated ALT levels were considered as active chronic carriers.

### Statistical analysis

Statistical analysis was performed using the statistical package for social studies (SPSS) version 16 for windows. Student t-test and chi-square tests were applied to evaluate differences in proportions. *P *value <0.05 was considered significant. Univariate analysis includes the variables age, HBV DNA levels, ALT and AST. Gender and PCR results were taken as independent categorical factors. Spearman correlation was used to assess the association between two quantitative variables. The diagnostic validity of serum ALT, AST and HBV DNA load and their combination were tested for classification of HBeAg (-) patients into active and inactive chronic patients. ROC curves were drawn for predicting values of ALT, AST and ALT at specific cut off values. Cutoff values for AST and ALT were 40 and 35 IU/L for each respectively, while for HBV DNA cut off values were 2000, 5000, 20,000, 50,000 and 100,000 copies/mL, respectively. New cut off values with high sensitivity, specificity, PPV and NPV were also predicted.

## Results

### Patient's clinical characteristics

A total of 567 HBeAg (-) patients were selected for this study. Patient's data is given in Table 1. The mean age of the patients was 32.20 ± 11.9. Of 567 patients, 228 were classified into HBeAg (-) chronic inactive, while remaining 339 were active. The age difference between both groups was not significant (*P *= 0.181). Male were dominant in both groups. Out of 228 inactive and 339 active patients, 58 and 114 were female respectively (*P *= 0.038). Among 170 chronic inactive male, 168 have ALT < 30IU/L; while 51 chronic inactive female out of 58, have ALT < 19 IU/L. Regarding AST levels, 225 inactive and 74 active carriers have their AST levels < 35 IU/L. Baseline ALT and AST values were significantly higher in HBeAg (-) chronic active patients (*P < 0.05*).

**Table 1:** Clinical characteristics of HBsAg positive patients

Patients characteristics	Inactive carriers n = 228	Active carriers n = 339	*P*-value
M/F	170/58	225/114	0.038
Age	31.3 ± 12.1	32.75 ± 11.7	0.181
ALT (IU/L)	17.3 ± 4.3	70.3 ± 15.1	0.000
AST (IU/L)	15.9 ± 7.1	48.4 ± 22.6	0.000
HBV DNA (copies/mL)	4.9 × 10^3^	6.5 × 10^8^	0.000

HBV DNA levels were five times elevated in active carriers (HBV DNA levels = 4.38 × 10^8 ^(± 1.04 × 10^9^) copies/mL *vs *6.9 × 10^4 ^(± 4.9 × 10^5^) copies/mL). More than 99% (n = 227) inactive carriers patients have HBV DNA levels less than 50, 000 copies/mL, and below undetected limits (< 200 copies/mL) in 21 patients.

### Application of revised cutoff values

**Figure 1:**
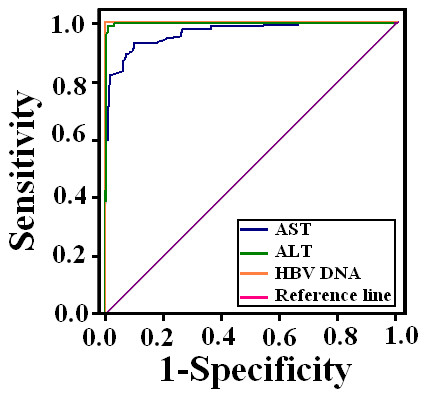
**ROC curve of serum ALT, AST and HBV DNA levels for HBeAg (-) patients showed serum ALT, AST and HBV levels could better predict HBV chronic active carriers at given cutoff value **.

Receiver operating characteristics (ROC) curves were drawn for ALT, AST and HBV DNA levels. All of them showed high area under the curve (AUC) to discriminate HBeAg (-) active carriers from inactive as mentioned in Table 2 (see also Figure 1).

**Table 2:** ROC curve analysis of serum AST, ALT and HBV DNA levels

Test Result Variable(s)	Area	SE	*P*-value	95% C I	
				
				Lower Bound	Upper Bound
**AST**	0.969	0.006	0.000	0.957	0.982
**ALT**	0.997	0.002	0.000	0.994	1.001
**HBV DNA level**	1.000	0.000	0.000	1.000	1.000

We observed approximately same sensitivity and specificity for the HBV DNA level ≥ 50,000 and NIH described limits ≥100,000 copies/mL. This value was much better than HBV DNA value of 2000, 5000 and 20,000 copies/mL. In general cohort, ALT and AST ≤ 20 IU/L were observed in 200 and 191 patients, respectively. ALT ≤ 30 IU/L showed high sensitivity (99.1%) and specificity (97.4%), while normal AST value (≤ 35 IU/L) showed high sensitivity (98.6%) but low specificity (77.8%) to discriminate active and inactive chronic HBeAg (-) carriers, respectively. In combination, ALT and HBV DNA levels; if ALT value were ≥30 IU/L for male and ≥19 IU/L for female, and HBV DNA load ≥100,000 copies/mL, a PPV of 97%, NPV of 94%, a sensitivity of 98%, and a specificity of 92% was observed to discriminate active carriers from inactive carriers (Table 3).

**Table 3:** Validity of serum ALT, AST and HBV DNA levels for the differentiation of patients with HBeAg (-) inactive chronic hepatitisB from active chronic HBeAg (-) carriers

Lab tests	Spe%	Sen%	PPV%	NPV%	Inactive carriers (n = 228)	Chronic carriers (n = 339)
**ALT (IU/L)**						
≤ 20	99.1	87.7	99	92	200/28	2/337
≤ 30	97.4	99.1	96	99.3	226/2	8/331
≤ 35	96.6	99.6	96	99.6	227/1	9/330
≤ 40	93.4	100	93	100	228/0	16/323
**AST (IU/L)**						
≤ 20	93.5	83.7	82	89.5	191/37	40/317
≤ 30	79.1	95.6	75.4	96.4	218/10	71/268
≤ 35	77.8	98.6	75.2	98.8	225/3	74/264
≤ 40	63.1	99.1	64.4	99.5	227/1	124/214
**HBV DNA (Copies/mL)**						
≤ 2000	100	48.2	100	75	110/118	0/339
≤ 5000	100	75.4	100	84	172/56	0/339
≤ 20,000	100	95.1	100	95	217/21	0/339
≤ 50,000	100	99.1	100	99	227/1	0/339
≤ 100,000	100	100	100	100	228/0	0/339
**ALT + HBV DNA (Our findings)**						
ALT (30 M/19F) HBV DNA (≤ 100,000 copies/mL)	92	98	97	94	210/18	5/334
**ALT + HBV DNA (Assy *et al. ***[22])						
ALT (30 M/19F) HBV DNA (≤ 100,000 copies/mL)	100	92	100	86	-	-

**Figure 2:**
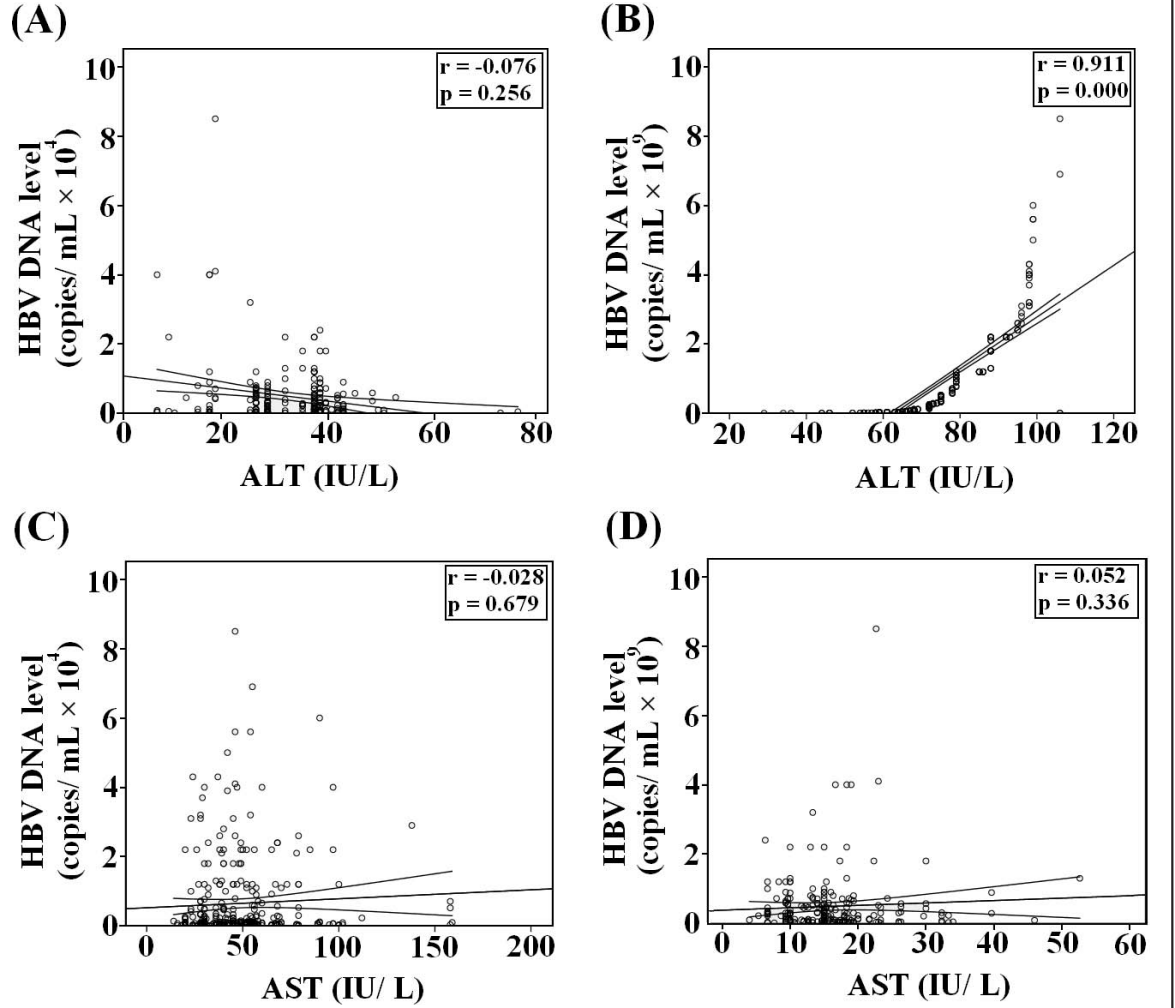
**Correlation of ALT and AST with HBV DNA levels in HBeAg chronic inactive and active patients **. A and C: association between ALT and AST with HBV DNA levels in chronic inactive carriers; B and D: association between ALT and AST with HBV DNA levels in chronic active carriers.

A statistical significant correlation was found between HBV DNA levels and ALT in HBeAg (-) chronic active patients (*r *= 0.911, *P < 0.05*). However, no such association was observed in case of ALT in chronic inactive patients and AST in both groups (Figure 2).

## Discussion

In this study, we assess the performance of new cut off values for serum ALT levels in male and female to predict active HBV in HBeAg (-) patients. As high ALT levels are thought to be associated with chronic HBV, and are commonly used during evaluation of HBV [10,25-28]. It is interesting to know that HBV evaluation depend on geographical association of the host and viral factors. Prati *et al. *proposed new cutoff value of ALT ≥ 30 IU/L in male and ≥19 IU/L in female [12], while Assy *et al. *2009 reported ALT ≥ 30 IU/L in male and ≥ 19 IU/L in female along with HBV DNA levels ≥ 100000 copies/mL can classify a patient into the active carrier state [22]. Although, serum AST levels are not thought to be incredibly useful predictor of HBV disease, we also evaluate their performance either they are useful or not for discriminating HBeAg (-) chronic active from inactive patients.

Previous studies reported raised serum ALT levels from ULN can predict liver dysfunction with 90% specificity and 56% sensitivity [29], but according to Kim *et al. *prior testing of ELISA along with ALT level can better predict liver function as compared to only ALT levels [30]. This is particularly important because without performing PCR and liver biopsy, the decision as to predict HBeAg (-) chronic inactive is difficult. ROC curve analysis was performed to find out an accurate cutoff value for ALT, AST and HBV DNA load to guess HBeAg (-) inactive chronic patients from active (Table 2 and 3). Serum ALT, AST and HBV DNA levels were found to be highly significant with immense AUROC. Their performance was assessed by using different cut off values irrespective of patient's gender. We observed same results as described by Prati *et al. *[12] and Assy *et al. *[23]. ALT ≤30 IU/L and HBV DNA load ≤ 100,000 copies/mL showed high sensitivity, specificity, PPV and NPV to differentiate HBeAg (-) inactive chronic patients from active. In combination (ALT and HBV DNA levels) we observed higher sensitivity (98%) and NPV (94%) than previously described [22] (92% and 86%, respectively) (Table 3).

Although Borg *et al. *found AST level as an important predictor during same circumstances [31], and in our study single AST value (≤ 20 IU/L) also showed high sensitivity and specificity, its prognostic ability was not better than serum ALT and HBV DNA. Our results are in agreement with Assay *et al *[22]. that the new baseline value for ALT levels (30 IU/L for male and 19 IU/L for female) notably perform well than AST as given in Table 3.

We detect HBV DNA in all patients. By using new cut off value of ALT, HBV DNA cut off values 50, 000 and 100,000 copies/mL showed same investigative performance and were better than 2000, 5000 and 20,000 copies/mL. These results indicate that NIH proposed HBV DNA levels limits are useful, and our findings are according to the study by Assy *et al. *[22] as given in Table 3. As HBV DNA load and liver damage appears to be different in HBeAg (+) and negative patients. In HBeAg (+) patients, no correlation was found between severity of liver damage and HBV DNA load [32-34]. In recent study by Kim *et al. *(2011) validate the performance of ALT and HBV DNA, and found that these markers may also used for discriminating patients with HBeAg (-) active carriers from inactive [35]. In our study, HBV DNA load was five times higher in chronic active patients. We also observed a positive significant correlation between HBV DNA levels and ALT in chronic active patients (Figure 2) leading to the conclusion that inflammation increases in patients with elevated HBV DNA levels as HBeAg has immunomodulatory action [36]. Recent studies showed that for HBeAg (-) patients, low HBV DNA levels are associated with less liver damage although some studies were unable to observe such relationship [37,38]. These findings suggest that HBV DNA load and ALT are most convenient techniques to predict active chronic HBV in HBeAg (-) patients.

Although, there are some limitations in our study like absence of liver biopsy data, HBV genotyping and/or a short period of follow up; yet the population size in this study is far larger than reported by others. In conclusion, we verified the new cut off value of ALT and found better results than previously described and also found AST as good predictor.

## Competing interests

The authors declare that they have no competing interests.

## Authors' contributions

BI, WA and FTJ contributed equally to this work. BI, WA, SG, FTJ and SH designed the study, analyze the data and wrote paper. They also checked the revised manuscript thoroughly and confirmed all the data given in manuscript. All work was performed under supervision of SH. We all authors read and approved the final manuscript.

## Authors' information

Bushra Ijaz (M Phil Molecular Biology), Waqar Ahmad (M Phil Chemistry) and Gull S (MSc Biochemistry) are Research Officer; Javed FT is Head Pathology Department Jinnah Hospital, Lahore; while Sajida Hassan (PhD Molecular Biology) is Principal Investigator at CEMB, University of the Punjab, Lahore
